# Supplementary material for: Novel Cold-Adapted Recombinant Laccase KbLcc1 from Kabatiella bupleuri G3 IBMiP as a Green Catalyst in Biotransformation
Source: Int J Mol Sci. 2021 Sep 4;22(17):9593. doi: 10.3390/ijms22179593 (PMC8431773; doi:10.3390/ijms22179593)
Supplement: Supplementary file 1 [file ijms-22-09593-s001.zip › ijms-1356440-supplementary.pdf]

Novel cold-adapted recombinant laccase KbLcc1 from *Kabatiella bupleuri* G3 IBMiP as a green catalyst in biotransformation

Katarzyna M. Wiśniewska, Aleksandra Twarda-Clapa and Aneta M. Białkowska*

SUPPLEMENTARY INFORMATION

Contents:

Table S1. Optimal temperature for laccase reaction with syringaldazine, guaiacol and sinapic acid 1
Table S2. Data to substrate specificity of laccase KbLcc1 – reaction with ABTS.................................. 2
Table S3. Data to substrate specificity of laccase KbLcc1 – reaction with syringaldazine...................... 2
Table S4. Data to substrate specificity of laccase KbLcc1 – reaction with guaiacol. 3
Table S5. Data to substrate specificity of laccase KbLcc1 – reaction with sinapic acid. 3
Figure S1. Influence of Cu^{2+} ions supplementation of expressing medium BMMY on recombinant laccase activity after 7 d of cultivation of Pichia pastoris 3
Figure S2. Stability of recombinant laccase KbLcc1 activity after 10 d of incubation in the presence of various metal ions. 4
Figure S3. GC-MS chromatograms of vanillin after 24 h biotransformation of ferulic acid by laccase KbLcc1.. 5
Figure S4. GC-MS chromatograms of vanillin after 48 h biotransformation of ferulic acid by laccase KbLcc1.. 6
Figure S5. GC-MS chromatograms of vanillin after 72 h biotransformation of ferulic acid by laccase KbLcc1.. 7
Figure S6. GC-MS chromatograms of vanillin after 96 h biotransformation of ferulic acid by laccase KbLcc1.. 8
Figure S7. GC-MS chromatograms of vanillin after 120 h biotransformation of ferulic acid by laccase KbLcc1.. 9

Table S1. Optimal temperature for laccase reaction with syringaldazine, guaiacol and sinapic acid.

Temperature (°C)	Residual activity ± SD (%)		
	Syringaldazine	Guaiacol	Sinapic acid
15	55.58 ± 0.79	61.55 ± 1.56	72.20 ± 0.90
20	63.22 ± 1.10	66.05 ± 1.56	85.77 ± 1.57
25	95.25 ± 1.53	86.51 ± 1.05	95.52 ± 0.92
30	100.00 ± 1.21	100.00 ± 1.07	100.00 ± 2.01
35	94.52 ± 1.22	93.22 ± 1.10	87.53 ± 0.96
40	88.31 ± 1.66	83.50 ± 1.51	75.03 ± 1.71
45	74.17 ± 0.99	63.09 ± 2.90	67.04 ± 1.68
50	54.47 ± 1.51	43.50 ± 1.54	47.97 ± 2.25

Table S2. Data to substrate specificity of laccase KbLcc1 – reaction with ABTS.

Substrate concentration (mM)	Activity (U/mg)			Mean ± SD (U/mg)
0.2	8.71	8.70	8.42	8.61 ± 0.17
0.4	15.41	14.27	15.29	14.99 ± 0.63
0.6	17.79	17.45	17.08	17.44 ± 0.35
0.8	19.84	19.34	19.09	19.43 ± 0.38
1.0	22.19	21.72	20.76	21.56 ± 0.73
1.2	22.19	23.47	23.75	23.13 ± 0.83
1.4	23.90	23.77	24.97	24.21 ± 0.66
1.6	24.19	23.95	24.66	24.27 ± 0.36
1.8	24.97	25.29	25.73	25.33 ± 0.38
2.0	25.72	26.45	27.13	26.37 ± 0.71
2.5	27.54	27.20	27.86	27.53 ± 0.33
3.0	28.14	29.44	29.07	28.88 ± 0.67
3.5	30.73	29.45	28.76	29.58 ± 1.03
4.0	29.13	29.59	30.21	29.64 ± 0.54

Table S3. Data to substrate specificity of laccase KbLcc1 – reaction with syringaldazine.

Substrate concentration (μM)	Activity (U/mg)			Mean ± SD (U/mg)
5.5	6.96	6.45	6.85	6.75 ± 0.27
6.0	7.20	7.49	6.93	7.21 ± 0.28
6.5	7.91	7.35	7.72	7.66 ± 0.29
7.0	8.36	7.98	8.02	8.12 ± 0.21
7.5	8.68	9.10	8.64	8.81 ± 0.26
8.0	9.54	8.95	9.27	9.25 ± 0.30
8.5	9.06	9.61	10.20	9.62 ± 0.56
9.0	9.37	10.28	10.42	10.02 ± 0.57
10.0	10.10	10.42	11.03	10.51 ± 0.47
15.0	13.55	14.06	13.85	13.82 ± 0.25
20.0	16.68	16.84	16.06	16.53 ± 0.41
25.0	17.23	17.27	17.99	17.50 ± 0.43
30.0	18.07	18.63	19.37	18.69 ± 0.65
35.0	20.02	20.36	20.78	20.40 ± 0.36
40.0	21.26	21.61	20.98	21.28 ± 0.32

Table S4. Data to substrate specificity of laccase KbLcc1 – reaction with guaiacol.

Substrate concentration (mM)	Activity (U/mg)			Mean ± SD (U/mg)
2.5	0.75	0.71	0.69	0.72 ± 0.03
5.0	1.37	1.42	1.36	1.38 ± 0.03
7.5	1.76	1.82	1.75	1.78 ± 0.04
10.0	2.12	2.13	2.07	2.11 ± 0.03
11.0	2.11	2.09	2.13	2.11 ± 0.02
12.0	2.19	2.20	2.17	2.19 ± 0.02
13.0	2.25	2.26	2.24	2.25 ± 0.01
14.0	2.37	2.42	2.33	2.37 ± 0.05
15.0	2.41	2.38	2.43	2.41 ± 0.03
16.0	2.1	2.51	2.57	2.53 ± 0.04
17.0	2.51	2.58	2.53	2.54 ± 0.03
18.0	2.67	2.70	2.64	2.67 ± 0.03
19.0	2.68	2.70	2.65	2.68 ± 0.02
20.0	2.62	2.67	2.71	2.67 ± 0.04

Table S5. Data to substrate specificity of laccase KbLcc1 – reaction with sinapic acid.

Substrate concentration (μM)	Activity (U/mg)			Mean ± SD (U/mg)
3.0	0.24	0.23	0.24	0.24 ± 0.01
6.0	0.38	0.36	0.39	0.38 ± 0.01
12.0	0.64	0.67	0.66	0.66 ± 0.01
24.0	1.03	1.08	0.99	1.03 ± 0.04
56.0	1.05	1.06	1.04	1.05 ± 0.01
100.0	1.09	1.10	1.10	1.10 ± 0.00
110.0	1.17	1.18	1.20	1.19 ± 0.01
120.0	1.23	1.24	1.22	1.23 ± 0.01
150.0	1.26	1.29	1.28	1.28 ± 0.02

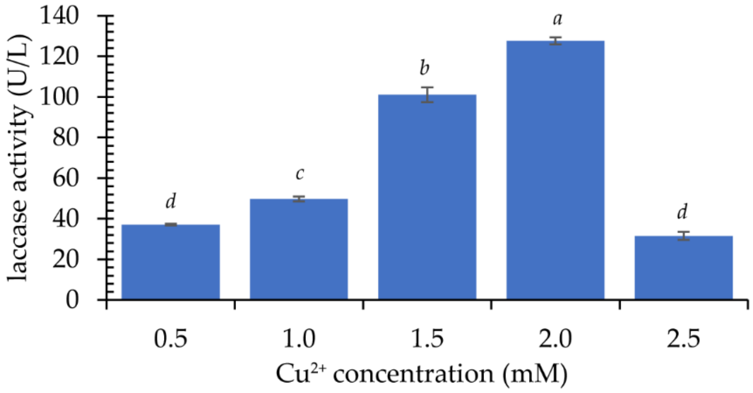

Figure S1. Influence of Cu^{2+} ions supplementation of expressing medium BMMY on recombinant laccase activity after 7 d of cultivation of *Pichia pastoris*.

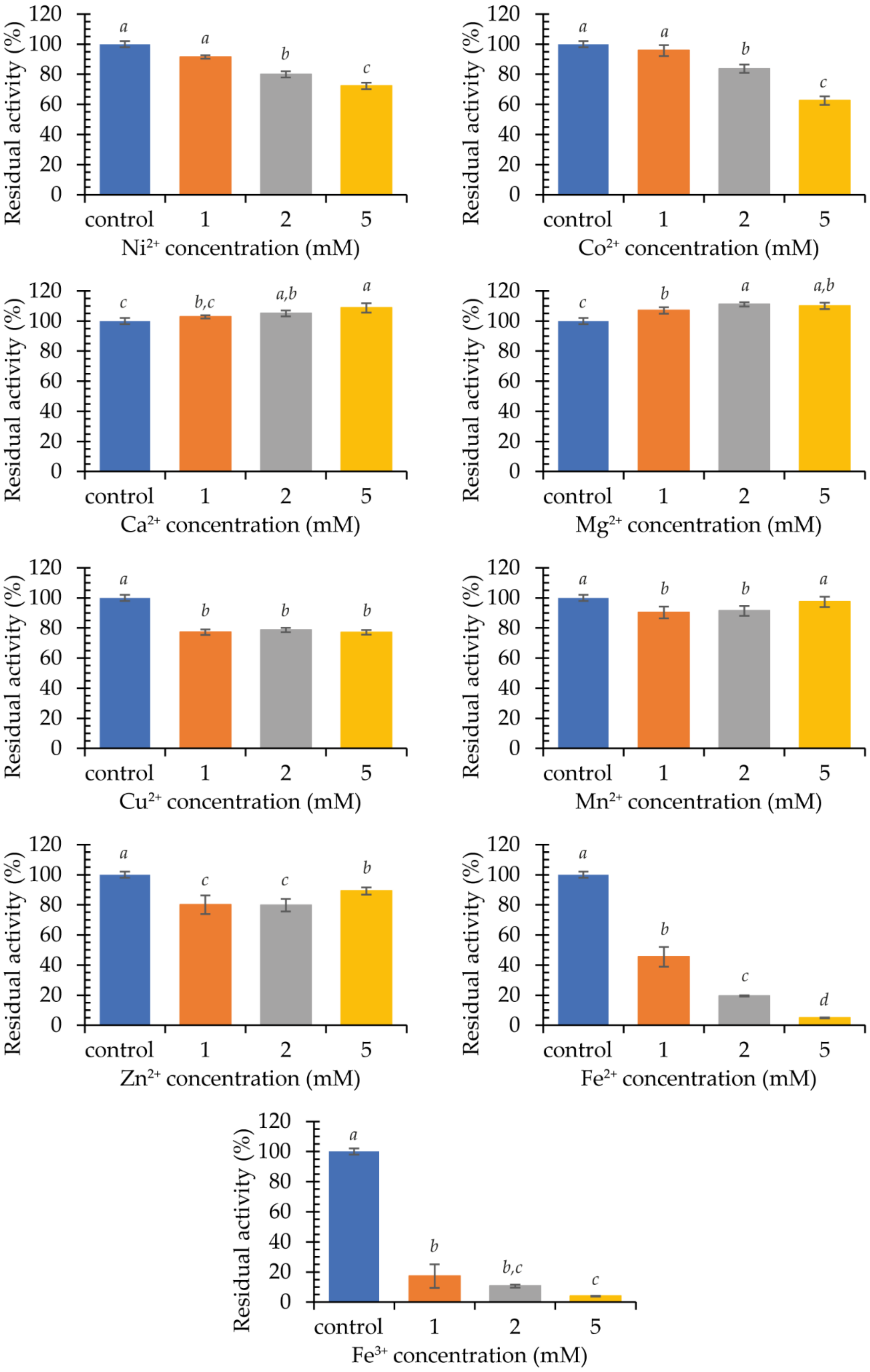

Figure S2. Stability of recombinant laccase KbLcc1 activity after 10 d of incubation in the presence of various metal ions.

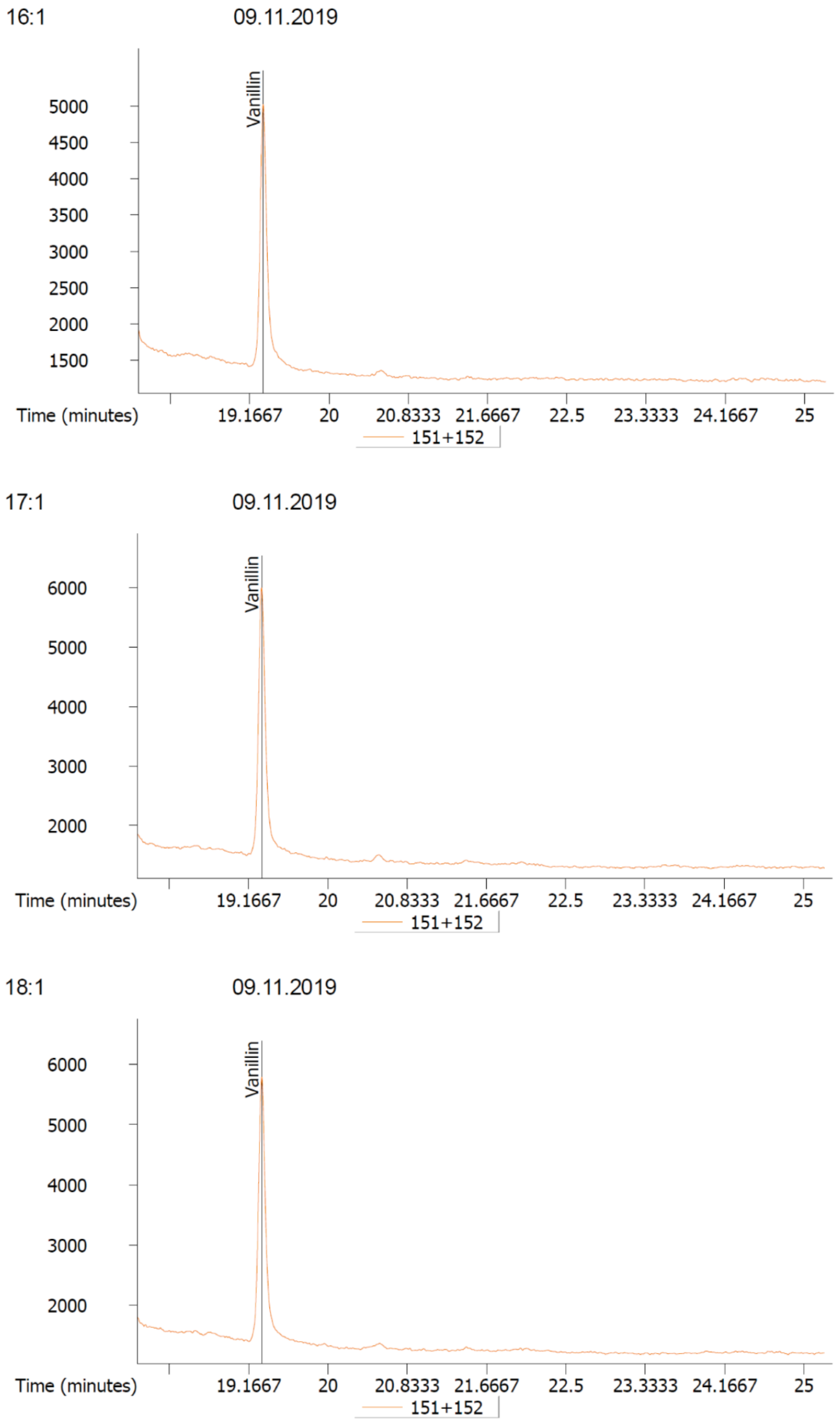

Figure S3. GC-MS chromatograms of vanillin after 24 h biotransformation of ferulic acid by laccase KbLcc1.

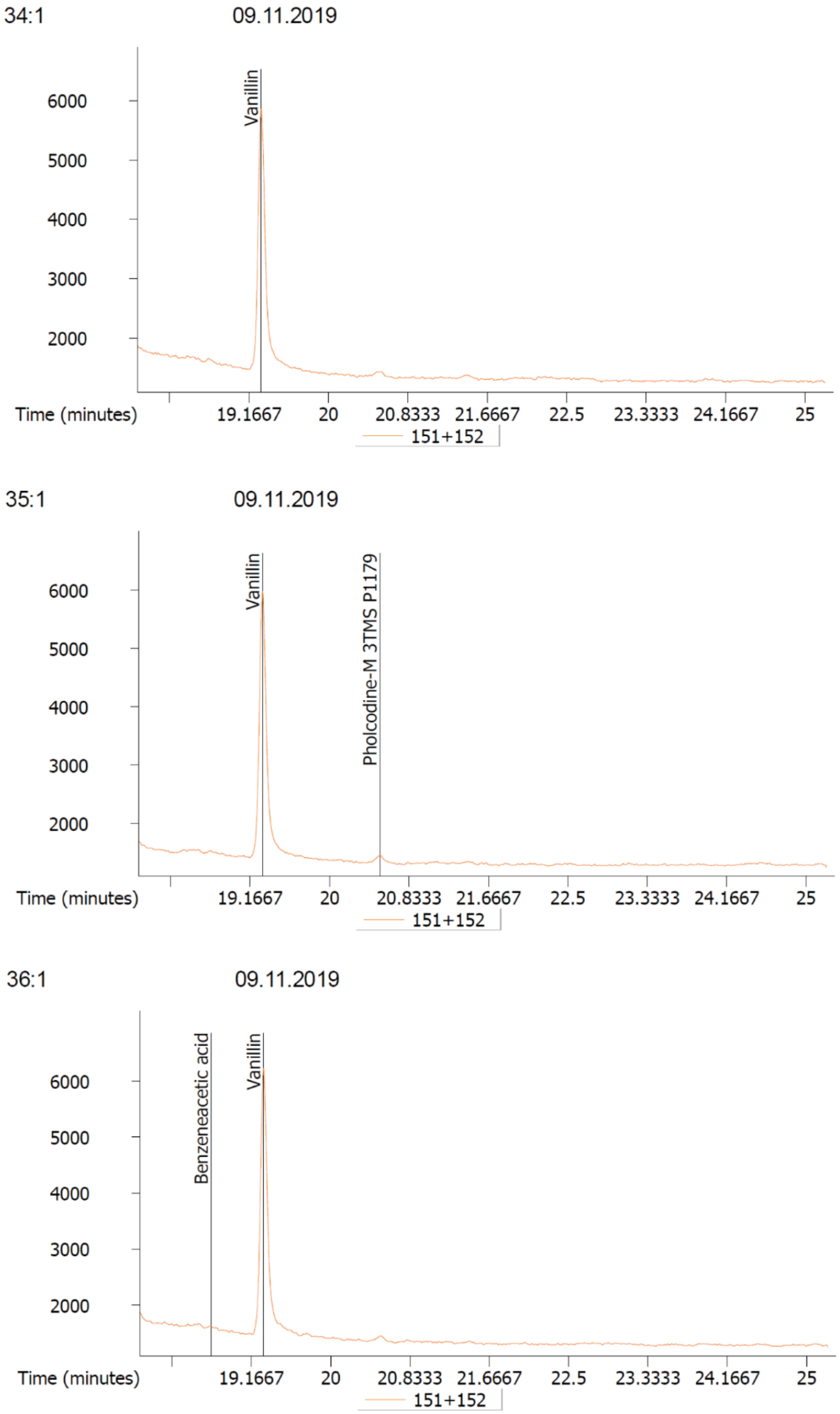

Figure S4. GC-MS chromatograms of vanillin after 48 h biotransformation of ferulic acid by laccase KbLcc1.

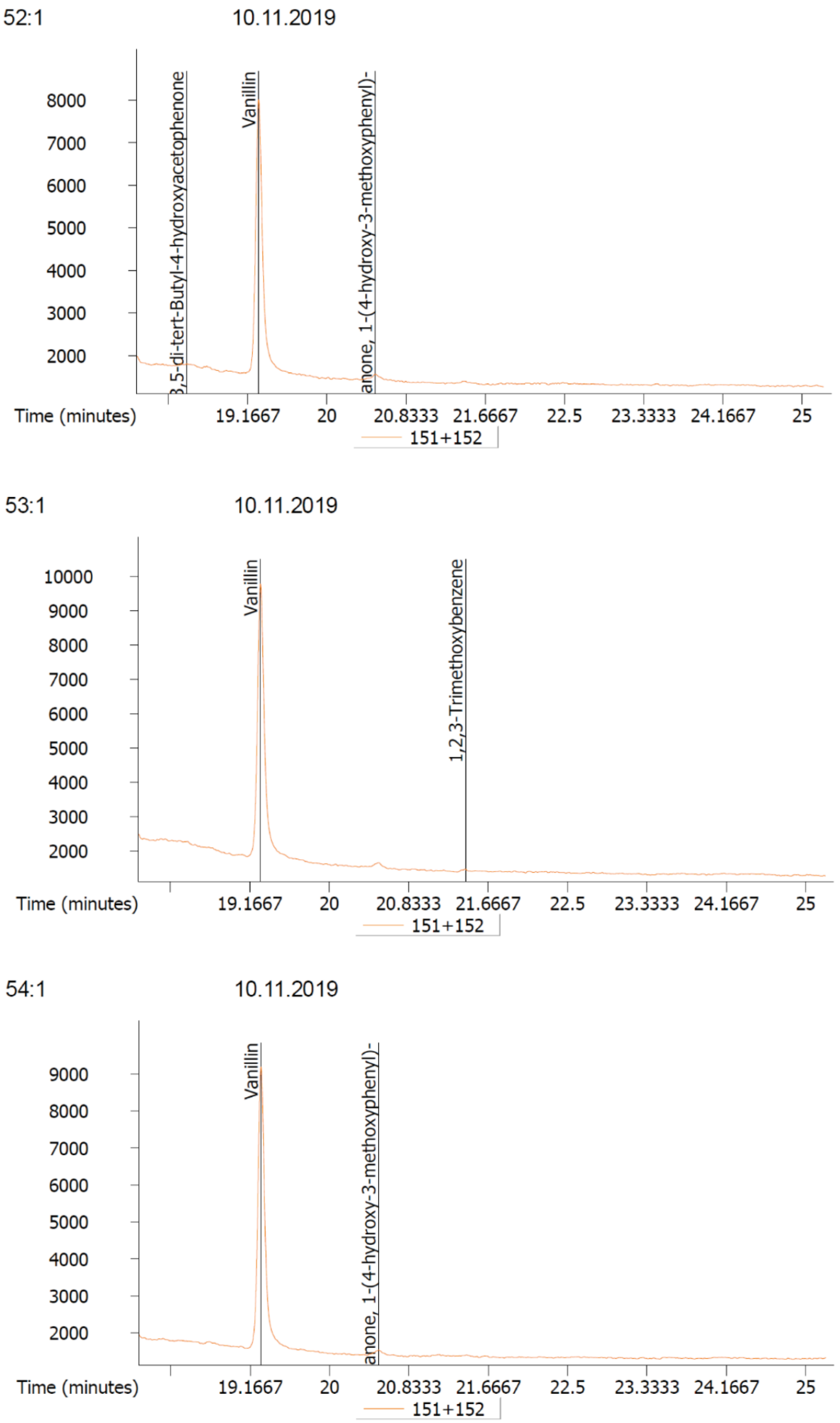

Figure S5. GC-MS chromatograms of vanillin after 72 h biotransformation of ferulic acid by laccase KbLcc1.

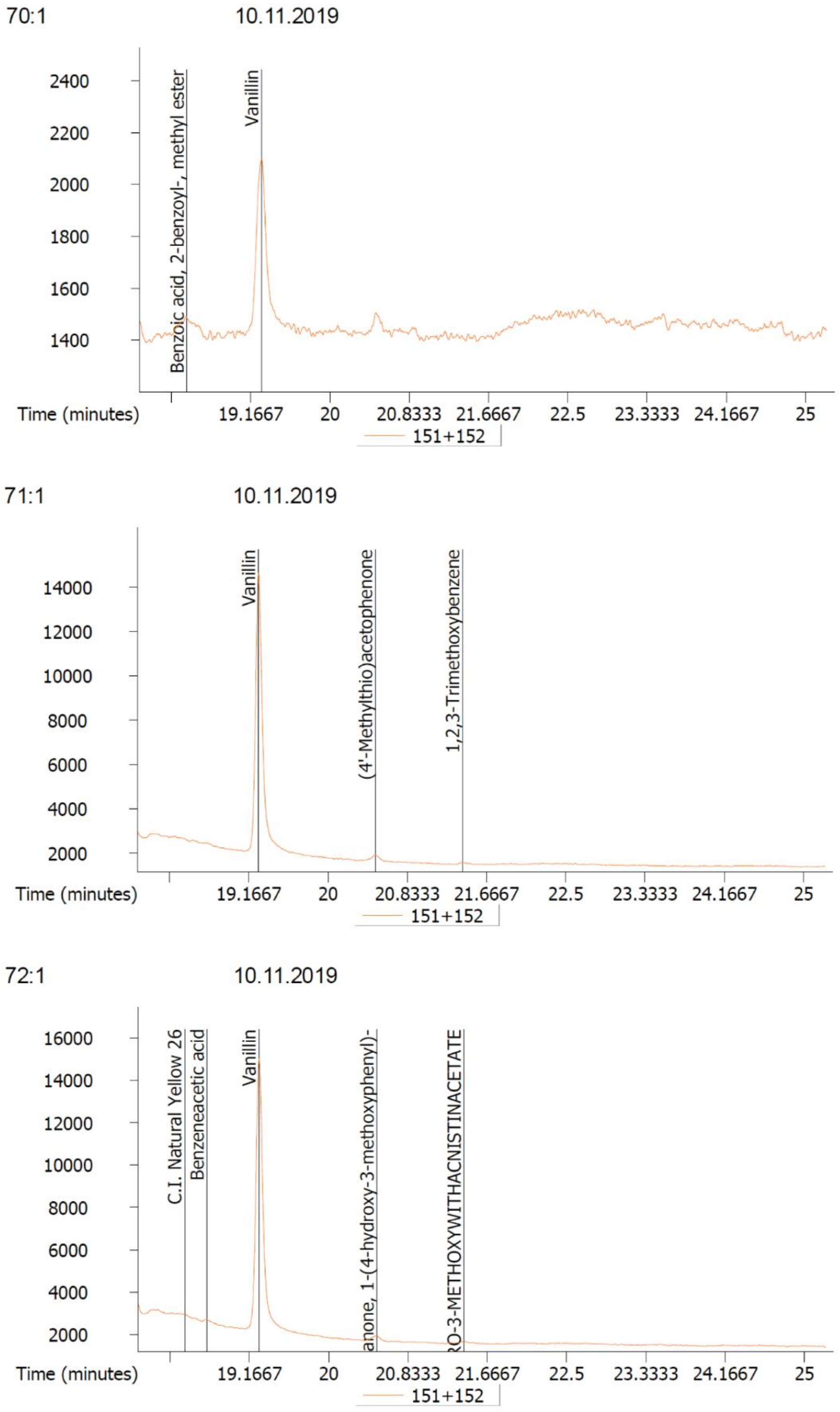

Figure S6. GC-MS chromatograms of vanillin after 96 h biotransformation of ferulic acid by laccase KbLcc1.

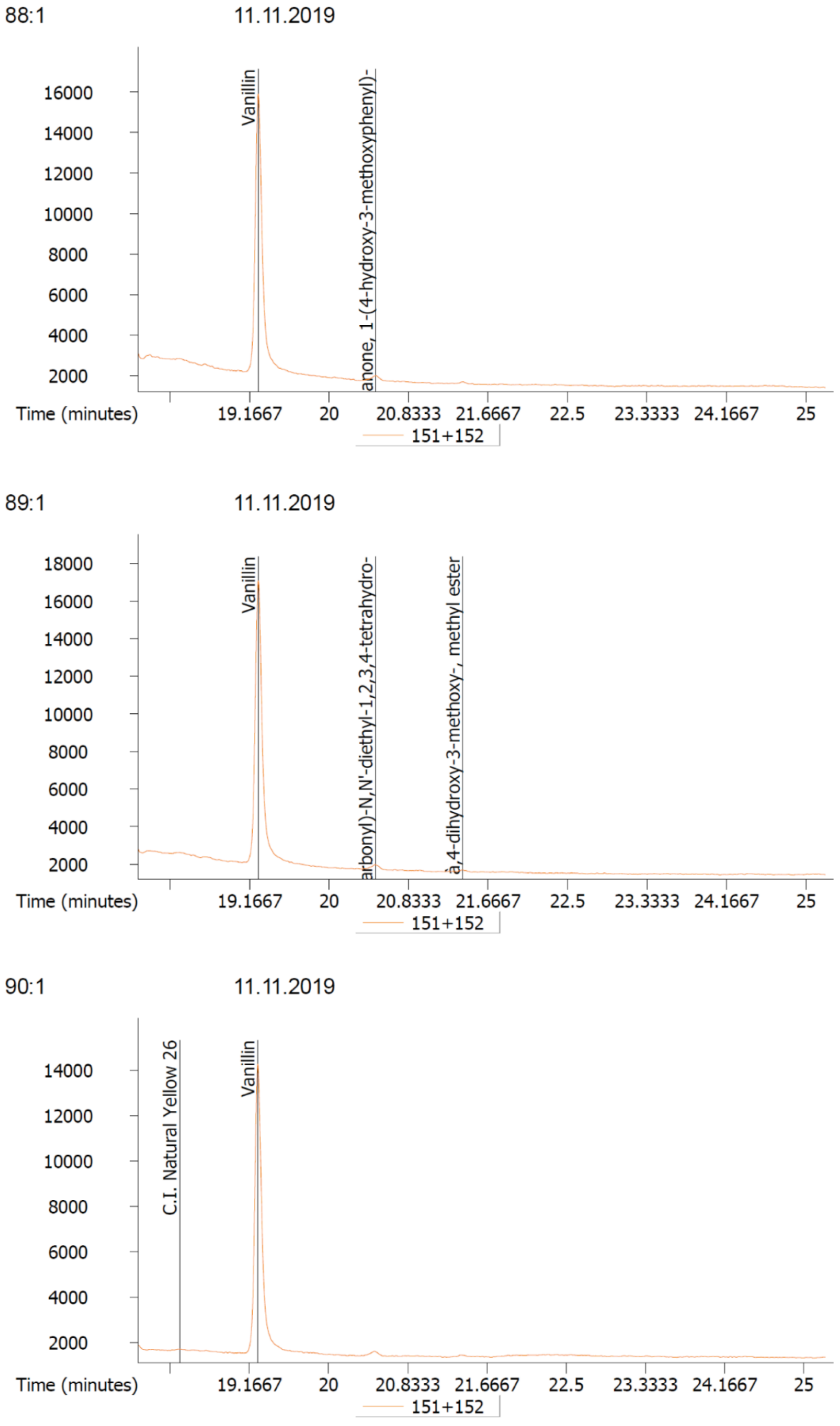

Figure S7. GC-MS chromatograms of vanillin after 120 h biotransformation of ferulic acid by laccase KbLcc1.
